# 

**Published:** 1915-09-11

**Authors:** 


### CONTENTS.

PAGE
**Tincture of Opium and Laudanum** ... ... 493
**The Medical Profession and the War Office...** 494
**Hospital and Institutional News ...** ... ... 495
A Lead from Liverpool—A Happy Inspiration—Honorary (War) Degrees—How Is It Done? Bob Sawyer at His Best—Poor-Law Officers Women in Military Hospitals—Sanatorium Benefit in Northampton—Infants and Drink—The British Association—Professor Schuster's Address—Humours of Typhus—The "Milne" Treatment Again—The Tyranny of the Regulation—Medical Students in War-Time—A Sanatorium and the Lighting Regulations—School Inspection at Ipswich—Insanity and the War—Tuberculous Sailors and Soldiers in Middlesex Medical Victims in the *Royal Edward* —The Supineness of Camberwell—The Nursing Difficulties in Infirmaries—No Room for the Mentally Deficient at Wandsworth—Fortunes in Proprietary Articles—Eucalyptus Once More—This Week's Drug Market—To Our Readers.
**Disabled Warriors and Parliament** ... ... 501
Their First Claim on the Nation's Resources.
**The Pneumococcus Carrier...** ... ... ... 502
**A Medical Causerie** ... ... ... ... ... 503

PAGE

**Round the World** ... ... ... ... ... 504

Fellow-Workers and the Roll of Honour.

**National Insurance Committees** ... ... ... 505

Injustice and Unreason.

**The Editor's Letter-Box** ... ... ... ... 506

An Appreciation of Mr. W. G. Carnt.

**Zeppelin Raids** ... ... ... ... ... ... 506

Fire Precautions at the London Hospital.

**The Heating of Hospitals** ... ... ... ... 507

II.—The Western Infirmary, Glasgow.

**Colliery Districts and Cardiff Hospital** ... 508

**The Institutional Library** ... ... ... ... 509

A Handbook for Parents—Tuberculosis Officers and Nurses.

**Endowed Beds with a History** ... ... ... 511

II.—In the Provincial and Scotch Hospitals.

**In the Law Courts** ... ... ... ... ... 512

Leicester Panel Doctor Sued.

**The Institutional Worker** ... ... (Suppl.) 1

The Purchase and Maintenance of Surgical Instruments and Appliances : Answer to August Question.

A Hospital's Publicity Department.

Enquiries and Answers.

